# Modelling the transmission and control strategies of varicella among school children in Shenzhen, China

**DOI:** 10.1371/journal.pone.0177514

**Published:** 2017-05-18

**Authors:** Xiujuan Tang, Shi Zhao, Alice P. Y. Chiu, Hanwu Ma, Xu Xie, Shujiang Mei, Dongfeng Kong, Yanmin Qin, Zhigao Chen, Xin Wang, Daihai He

**Affiliations:** 1 Shenzhen Center for Disease Control and Prevention, Shenzhen, China; 2 Department of Applied Mathematics, Hong Kong Polytechnic University, Hong Kong, China; Shanxi University, CHINA

## Abstract

**Objectives:**

Varicella (chickenpox) is a highly transmissible childhood disease. Between 2010 and 2015, it displayed two epidemic waves annually among school populations in Shenzhen, China. However, their transmission dynamics remain unclear and there is no school-based vaccination programme in Shenzhen to-date. In this study, we developed a mathematical model to compare a school-based vaccination intervention scenario with a baseline (i.e. no intervention) scenario.

**Methods:**

Data on varicella reported cases were downloaded from the Infectious Disease Reporting Information Management System. We obtained the population size, age structure of children aged 15 or under, the class and school distribution from Shenzhen Education Bureau. We developed an Agent-Based Susceptible-Exposed-Infectious-Recovered (ABM-SEIR) Model that considered within-class, class-to-class and out-of-school transmission modes. The intervention scenario was that school-wide vaccination intervention occurred when an outbreak threshold was reached within a school. We varied this threshold level from five to ten cases. We compared the reduction of disease outbreak size and estimated the key epidemiological parameters under the intervention strategy.

**Results:**

Our ABM-SEIR model provided a good model fit to the two annual varicella epidemic waves from 2013 to 2015. The transmission dynamics displayed strong seasonality. Our results suggested that a school-based vaccination strategy could effectively prevent large outbreaks at different thresholds.

**Conclusions:**

There was a considerable increase in reported varicella cases from 2013 to 2015 in Shenzhen. Our modelling study provided important theoretical support for disease control decision making during school outbreaks and the development of a school-based vaccination programme.

## Introduction

Varicella (chickenpox) is caused by the varicella zoster virus (VZV) of the Herpesviridae family. It spreads by direct contact and airborne droplets [1]. Varicella is highly transmissible during childhood, thus it has the potential to cause outbreaks at schools [1].

In China, varicella outbreaks pose serious public health threats to the school populations. The National Immunization Program does not cover vaccination against varicella, and they are only available as self-paid vaccines for children between one and 12 years of age [2]. Varicella uptake rate remains low in China, and most children only receive a single-dose vaccine [3], which, according to a recent meta-analysis, is only about 81% effective [4]. In October 2015, China introduced a two-child policy to replace its one child policy [5]. This policy change has led to increase in fertility rate and is expected to increase the future size of school populations. Thus, it is imminent to examine public health control strategies of varicella among schools in Shenzhen, China.

Transmission dynamics of infectious diseases had been investigated in previous studies. [6–9]. Several studies had explored the impact of vaccination on varicella transmission [10–13]. These studies applied a Who-Acquired-Infection-from-Whom (WAIFW) contact matrix, combined with age-specific transmission parameter following the methodology of Wallinga *et al.* [14], was primarily used empirical age-specific social contact data of European populations but failed to account for the class and school structure of student populations. Jackson *et al.* developed two mathematical models to study the effects of school holidays on the spread of varicella, and found that there were 22% to 31% reduction in student contacts during summer holidays, that led to a lower rate of varicella transmission [15]. A surveillance study were conducted at elementary schools, found school nurse surveillance and tracking of varicella cases are effective in lowering annual varicella incident cases [16].

In this study, we modelled the transmission dynamics of varicella among school children in 2013-2015 in Shenzhen, China. We considered two scenarios: (i) baseline (no intervention) scenario; (ii) school-based vaccination scenario, where all students within a school were vaccinated once the number of varicella cases were beyond a stated threshold. Here, an Agent-Based Susceptible-Exposed-Infectious-Recovered(ABM-SEIR) Model was developed, and we showed that reasonable modelling estimates could be achieved with this model by specifying individual level and group-level contact patterns [17–19]. This paper is structured as follows: First, we described the data source, model population and model structure. Next, we described the model parameters and model fitting. It is then followed by the estimation of reproduction number under different intervention scenarios. Lastly, we discussed the implications of our findings.

## Data and methods

### Data

**Fig 1:**
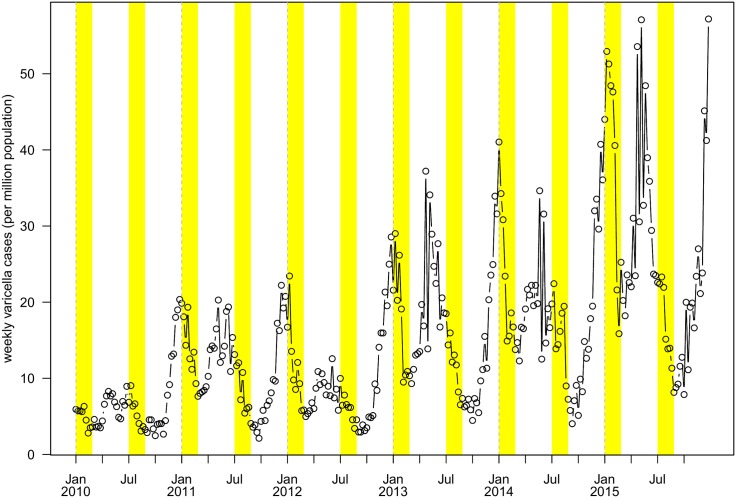
Number of weekly varicella confirmations from 2010 to 2015 per 1,000,000 population in Shenzhen from 2013 to 2015. Weekly population is computed using *loess* model. School holidays are shaded in yellow.

The varicella reported cases were obtained from Infectious Disease Reporting Information Management System in Shenzhen, China, on a weekly basis from 2010 to 2015. These reported cases included both clinically diagnosed cases and laboratory confirmed cases, which were voluntarily reported by local medical doctors. In Fig 1, we show the weekly reported cases per 1,000,000 population, and an increasing trend is observed from 2013 to 2015. The reported cases show a peak-to-trough pattern from school terms to school holidays. We also collected data on the monthly total number of school outbreaks from January 1, 2010 to December 31, 2015 from Shenzhen Center for Disease Control and Prevention (SZCDC). The school outbreaks were reported under a compulsory surveillance system. A school outbreak is defined as five or more varicella cases within a seven day period that occurs at a school or kindergarten.

**Fig 2:**
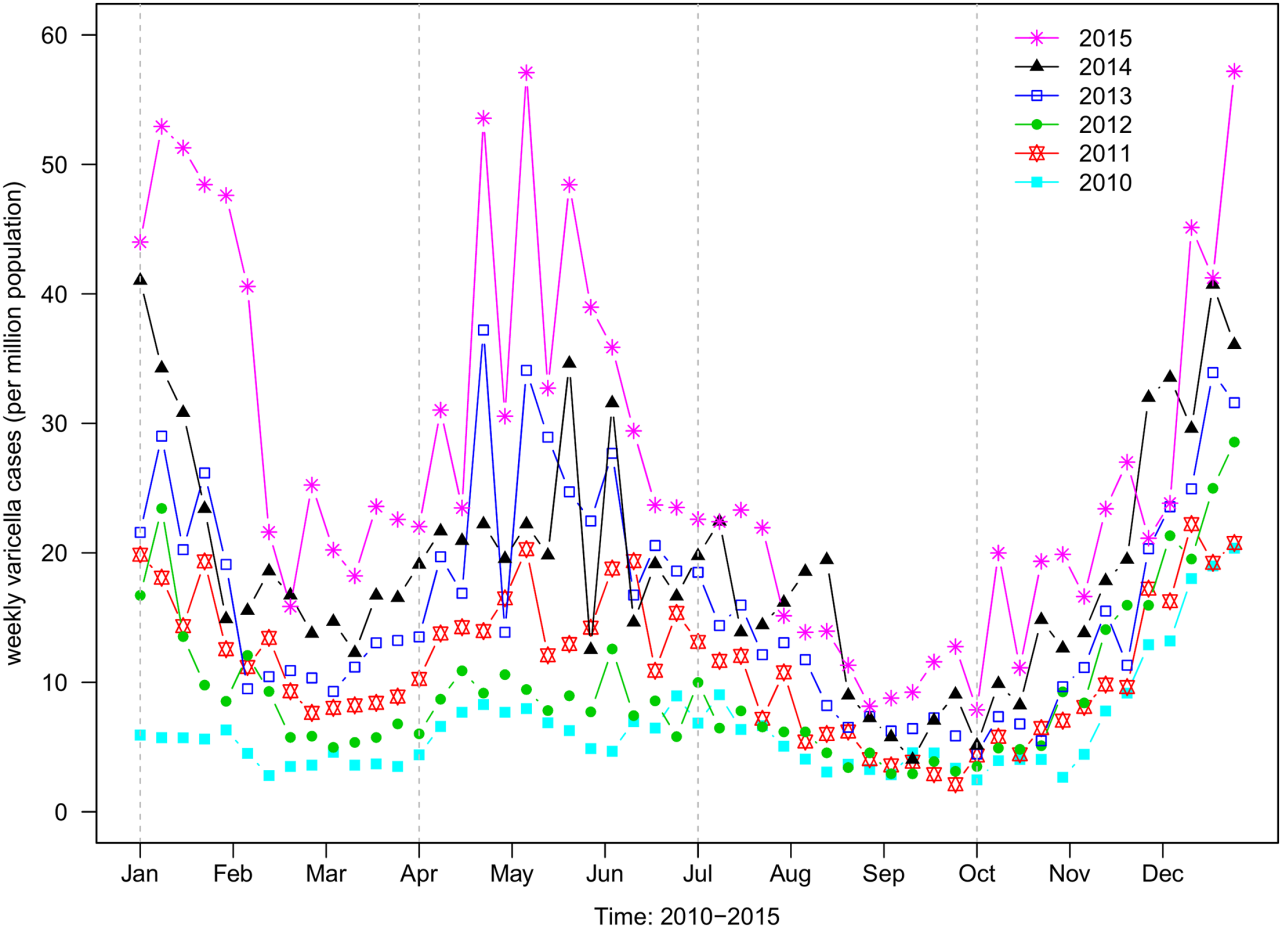
Weekly number of varicella confirmations in each year from 2010 to 2015, per 1,000,000 population. Weekly varicella cases is computed using *LOESS* model.

In Fig 2, we used a locally weighted scatterplot smoothing model (LOESS) to show the monthly school outbreaks from 2010 to 2015. We found that both reported cases and school outbreaks displayed two epidemic waves annually. Apparently, the trend in weekly reported cases lagged behind the school outbreaks.

**Fig 3:**
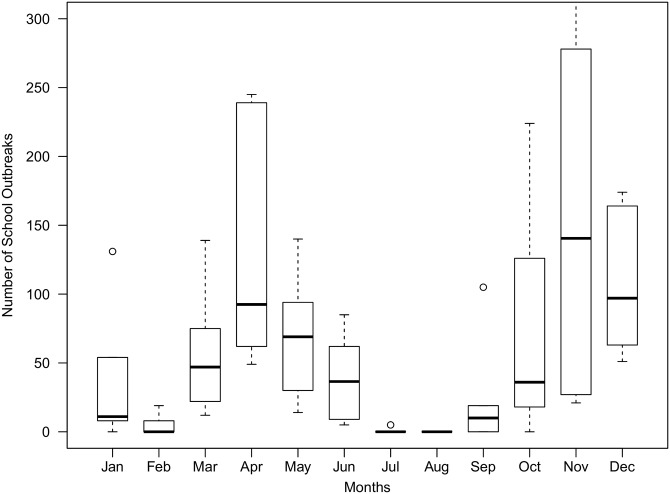
Boxplot of the number of varicella school outbreaks from 2010 to 2015, which displays similar patterns as in Fig 2. The number of school outbreaks per 30 days is displayed, to adjust for the variations of the number of days in each month.

In Fig 3, we show the number of varicella school outbreaks from 2010 to 2015 in a boxplot. We could see that there are two epidemic peaks in April and November and a trough in July and August annually.

**Fig 4:**
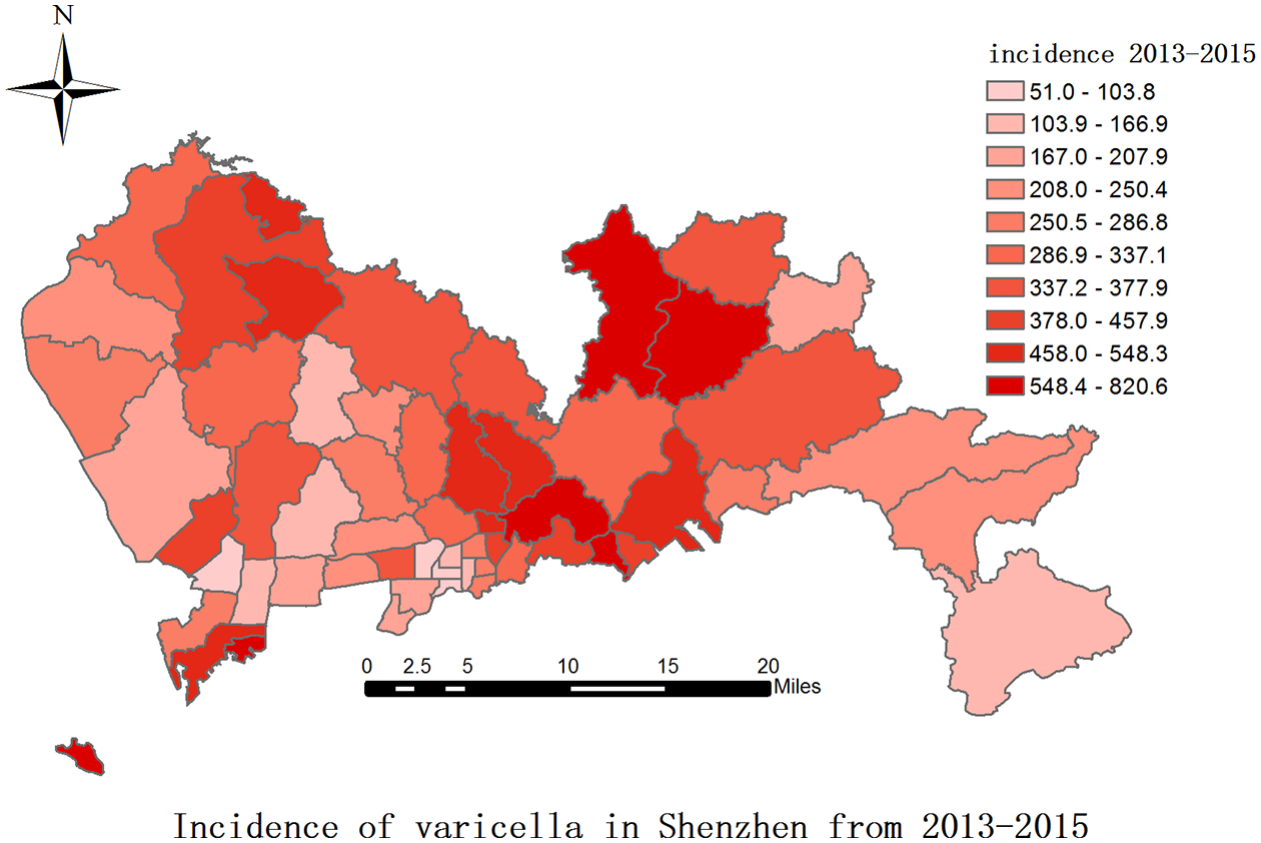
Varicella incidence distribution in Shenzhen by district from 2013 to 2015. The shade represents the levels of varicella incidence, cases are per 100,000 population within each district.

Fig 4 shows the distribution of varicella incidence in different districts from 2013 to 2015. We observe higher varicella incidence near Luohu which borders Hong Kong. There were substantial geographical variations. Information about the population size of those ages under 15, age structure and distribution of the number of classes and schools were downloaded from the website of Shenzhen Education Bureau [20].

### Target population

Our model population consisted of individuals from 0 to 15 years old in Shenzhen, as varicella primarily affects this age range. We did not consider the effects due to Herpes Zoster caused by VZV because of its extremely low incidence within this age range. For the school populations and the students’ age, class and school structure, we made adjustments including approximation and averaging to the official data [20]. Table 1 displays the distribution of the adjusted number of schools, classes and students by types of school.

**Table 1:** A summary table of the adjusted average number of schools, classes, and distribution of students in Shenzhen. Students per class, *N*_*j*,*i*_, was given by Eq (2)). The information was obtained from Shenzhen Education Bureau [20].

Age groups (years)	0 - 3	4 - 6	7 - 12	13 - 15
Student status	Pre-school	Kindergarten	Primary school	Secondary school
Schools in Shenzhen	300	1500	550	250
Classes per school	25	10	24	20
Students per class: *N*_*j*,*i*_	20	30	45	60
Proportion: *p*_*j*_	0.1004	0.3012	0.3976	0.2008

Note: The age group from 0 to 3 years consist of mainly pre-school children. Therefore, “schools in Shenzhen” actually reflects the number of street blocks, “classes in school” represent the number of communities per street block and “students per class” refers to the number of children in that age group within each community [18].

### Model structure

We developed an ABM-SEIR model for school students in Shenzhen. The overall model structure could be conceptualized as follows: students are nested within classes, classes within schools, and schools within Shenzhen’s school students population. A classical SEIR compartmental model was fitted to each class, while considering the different age structures and grade levels.

$$\begin{array}{c} \text{Students}\in \text{Classes}\subset \text{Schools}\subset \text{Shenzhen} \end{array}$$

#### Within-class transmission

As there were frequent social contacts and interactions with other classmates during a school day, each class was treated as a group-level unit for human-to-human transmission. Thus we applied a SEIR model to each class, and the classes were expressed as the following set of non-linear ordinary differential equations (ODE):

$$\begin{array}{ccc} \frac{dS}{dt} & = & -\theta (a)\cdot \beta SI\\ \frac{dE}{dt} & = & \theta (a)\cdot \beta SI-\sigma E\\ \frac{dI}{dt} & = & (1-\eta )\cdot \sigma E-\gamma I\\ \frac{dR}{dt} & = & \eta \sigma E+\gamma I \end{array}$$ (1)

Here, *S*, *E*, *I* and *R* denoted the number of Susceptible, Exposed, Infected and Recovered individuals respectively. The total number of students in each class was given by:

$$\begin{array}{c} N_{j,i}=S+E+I+R \end{array}$$ (2)

where, *j* denotes the *j*th school and *i* denoted the *i*th class within the *j*th school.

The other parameters were as follows: average transmission rate (*β*), average infectious rate (*σ*), average recovery rate (*γ*) and beta multiplier (*θ*(*a*)), the last of which was dependent on the student’s age (a). *η* was the average rate of losing infectiousness due to hospitalization, medical treatment or contact isolation [21].

We did not consider birth and death processes in the model, since our study period was relatively short compared with the average lifespan. The epidemiological effects of seasonal oscillations in birth rates were negligible [22]. Furthermore, once a student recovers from varicella, he or she would be immunized for 20 to 40 years, which is much longer than our study period. In effect, students entering the Recovered (*R*) compartment left the system.

#### Class-to-class transmission

The next level of transmission would be class-to-class varicella transmission which involved social contacts and mixing of students between different classes. Such activities include assembly gathering, having meals at school canteens, taking school buses and attending extra-curricular activities. We adopted the same operational definition by SZCDC and previous studies [3, 11, 16], where an outbreak threshold was reached when there were five or more varicella cases within a class, i.e. *I*_limit_ = 5 cases.

The spread rate, *δ*, between classes was low under current disease control measures. Otherwise, large outbreaks could occur among school populations.

#### Out-of-school transmission

**Fig 5:**
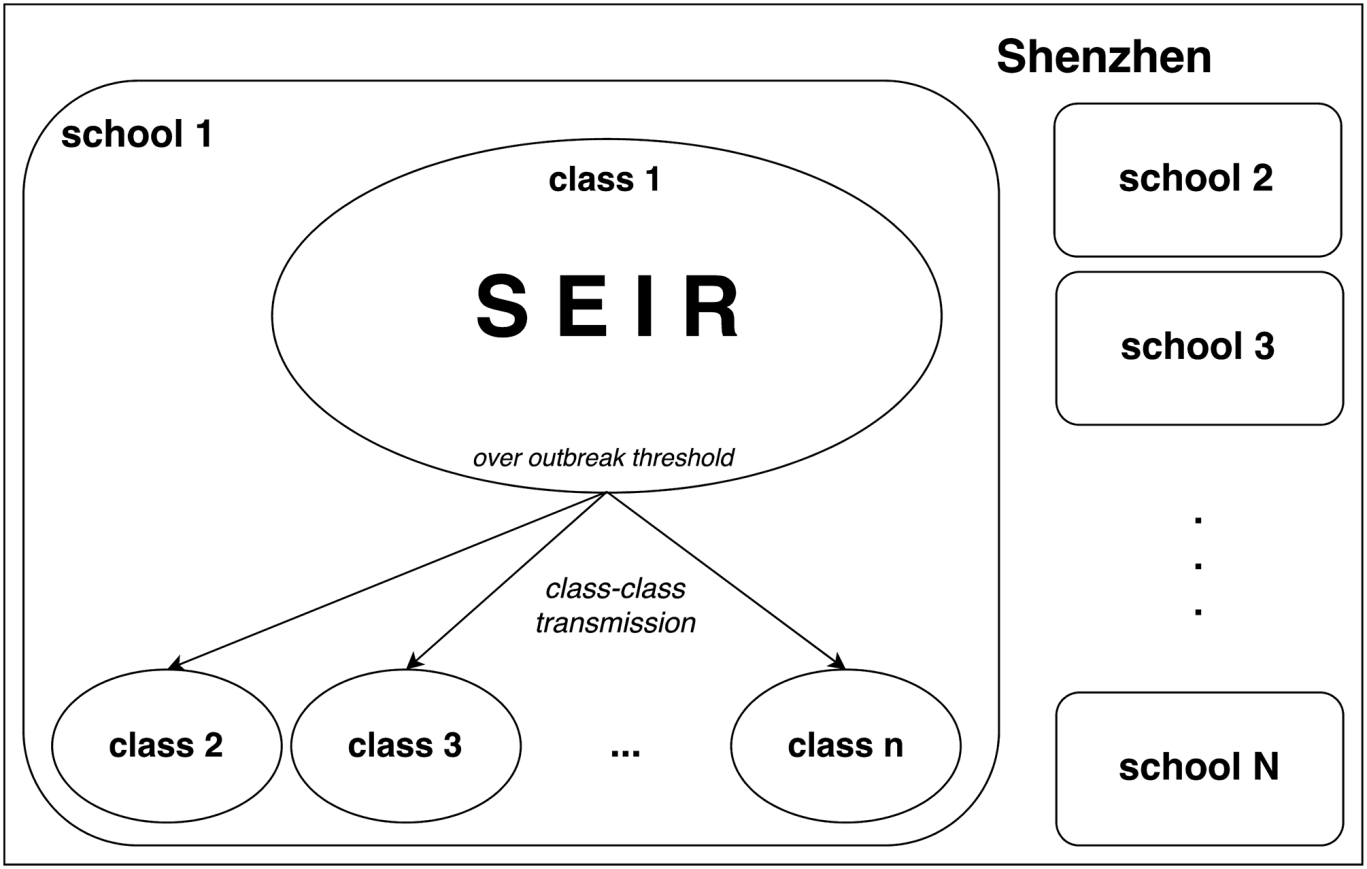
The structural diagram of the ABM-SEIR in Shenzhen. Within each classes, SEIR model structure is applied (see ODE System (1)). Within a school, if a class reaches the pre-defined outbreak threshold, there will be possible disease transmission to non-outbreak classes, to which we name “class-class transmission”. This transmission will vanish whenever the number of cases in the outbreak classes becomes lower than the outbreak threshold.

The third level of transmission would be out-of-school transmission due to student contacts between different schools. Inter-school activities and private group tutorials would be examples of such. Also, Shenzhen is a popular city with many tourists, businessmen and students visiting every year. Thus it was important to consider imported varicella cases to Shenzhen. However, the imported rate, *τ*, would be relatively low because out-of-school transmission was not a predominant transmission route in our model. Fig 2 shows obvious annual periodicity of the weekly reported varicella cases. These patterns were especially remarkable from 2013 to 2015. Previous studies attributed these to the seasonality of school terms [17, 23, 24]. We incorporated this factor in to the ABM-SEIR. Fig 5 shows the schematic diagram for the ABM-SEIR describing within-class transmission, class-to-class transmission and out-of-school transmission within Shenzhen’s school age populations.

### Model parameters

Del Fava *et al.* [25] found that varicella transmissibility would be the strongest within the youngest age groups. Thus we applied a beta multiplier (*θ*) to represent the relative transmissibility within each age group (See Table 2)

**Table 2:** Table of beta (or *β*) multiplier (*θ*) with respect to different age groups.

Age groups (year)	0 - 3	4 - 6	7 - 12	13 - 15
beta multiplier (*θ*)	0.625	1.000	0.750	0.500

The transmission rate, *β*, is defined as the probability of a susceptible to be infected after an effective contact with one infectious individual. This is a time-dependent function. Class-to-class transmission rate, *δ*, is estimated according to the best-fitted varicella transmission model. Table 3 summarizes the list of model parameters:

**Table 3:** Summary table of parameters.

Parameter	Notation	Value	Source
Latent period	*σ*^−1^	14 (day)	[26]
Infectious period	*γ*^−1^	7 (day)	[26]
Transmission rate	*β*	to be estimated	-
Beta multiplier	*θ*	Table 2	[25]
Initial immune percentage	*R*_0_	65.00%	[10]
Initial infectious percentage	*I*_0_	0.05%	[21]
Initial exposed percentage	*E*_0_	0.00%	assumed
Initial susceptible percentage	*S*_0_	34.95%	[1 − (*E*_0_ + *I*_0_ + *R*_0_)]
Class-to-class transmission rate	*δ*	to be estimated	-
Ratio of school cases to total cases	*ρ*	90.00%	[10, 12, 27]
Importing rate	*τ*	5.00%	assumed
Rate of losing infectiousness	*η*	30.00%	[21]

We initialize the ODE system (see Eq (1)) with the following values:

$$\begin{array}{c} \left\{S_{0},E_{0},I_{0},R_{0}\right\}=\left\{34.95\%,0.00\%,0.05\%,65.00\%\right\} \end{array}$$

According to SZCDC [3], it was common for children to be vaccinated or recovered from a varicella episode before entering schools, thus we assumed *R*_0_ to be 65%. As in Lenne *et al.*, we assumed the rate of losing infectiousness to be *η* = 30% [21]. This rate represented the losses due to medication, contact isolation and/or hospitalization.

### Model fitting

#### Fitting transmission rate

We proposed a continuous linear structure to our beta function:

$$\begin{array}{c} \beta \left(t_{\text{week}}\right)=c_{i}^{{}^{\prime }}+k_{i}\cdot t_{\text{week}} \end{array}$$

where, *β*(*t*_week_) was the transmission rate function, $c_{i}^{{}^{\prime }}$ was the constant term, *k*_*i*_ was the slope and *t*_week_ was the week number of the current year. The subscript *i* represented the *i*th week segment of the school term, which was segregated by two longer school holidays. In China, summer breaks last for two months from July to August. Winter breaks are usually from mid-January to mid-February, and takes place around the lunar new year. For convenience, we converted the beta function into the following form:

$$\begin{array}{c} \beta \left(t_{\text{week}}\right)=c_{i}+k_{i}\left(t_{\text{week}}-t_{i}\right)\;i\in \left\{1,2,3,...,M\right\}\;\&\;t_{\text{week}}\in \left[t_{i},t_{i+1}\right) \end{array}$$ (3)

where *t*_*i*_ is the starting week number of the *i*th week segment in current year and there are total *M* week segments in the current year. Since the beta function was continuous within a year, our model only needed to fit the constant term (*c*_*i*_) at the start of each week segment, i.e. node, such that, for the *i*th week segment, the estimated slope is given by:

$$\begin{array}{c} \hat{k_{i}}=\frac{{\hat{c}}_{i+1}-{\hat{c}}_{i}}{t_{i+1}-t_{i}} \end{array}$$ (4)

where $\hat{c_{i}}$ represents the fitted constant term for the *i*th week segment and the *t*_*i*_ is the starting week number of the *i*th week segment.

For each of the *M* nodes (*c*_*i*_), we assumed they were ranged between 0.00 and 0.50. The Monte Carlo (MC) method was applied to estimate the best-fitted $\hat{c_{i}}$ which had the smallest mean squared error.

#### School terms

In our model, we divided each school year into *M* segments (Eq (3)) according to the school calendar in China: within a school year there were two semesters, each containing three segments: school vacation, beginning of semester, and end of semester, resulting in a total of six segments per year, i.e. *M* = 6.

The transmission rate (*β*) between a school term and vacation were different due to the differences in contact frequencies and patterns [18, 23, 24]. We separated each school term into two segments for two reasons: (i) decrease in contact between the susceptible and infected on the onset of an outbreak; and (ii) difference in seasonality due to climatic factors such as temperature. Both reasons could lead to a change in the beta function.

#### Model simulation

We ran the simulation 1000 times for each node (or *c*_*i*_) combination. The Mean Squared Error (MSE) was the model fitting criteria between the weekly reported cases number and the model simulation median. A small number of cases in 2011 and 2012 were ignored, and the ABM-SEIR was only fitted to the reported cases from 2013 to 2015. The algorithm of parameter estimation were described in more details in S1 File.

The total number of infection cases, *N*, in Shenzhen, was given by:

$$\begin{array}{c} N=\frac{\sum_{j}\sum_{i}N_{j,i}}{\rho } \end{array}$$ (5)

where, *N*_*j*,*i*_ was given by Eq (2). The *c*_*i*_s’ combination with the smallest MSE was selected as the best-fitting model, and was adapted to the school-based vaccination scenario.

R software (version 3.3.1.) and Java (version 8) were used for modelling and computations.

### Estimation of basic reproduction number

Within our ABM, an infectious individual at model initialization could induce three levels of transmission. For the *j*th age group, the reproduction number for within class transmission is ${R_{\text{class}}}_{j}$:

$$\begin{array}{c} {R_{\text{class}}}_{j}=\frac{\left(1-\eta \right)\cdot \theta_{j}\bar{\beta }S_{0}n_{j}}{\gamma } \end{array}$$ (6)

The reproduction number for class-to-class transmission, ${R_{\text{c-c}}}_{j}$, is:

$$\begin{array}{c} {R_{\text{c-c}}}_{j}=\frac{\hat{\delta }\cdot \left({N_{\text{class}}}_{j}-1\right)\cdot \text{Pr}\left(I_{j}⩾I_{\text{limit}}\right)}{\gamma } \end{array}$$ (7)

For out-of-school transmission, or imported cases, the reproduction number, ${R_{\text{import}}}_{j}$, is:

$$\begin{array}{ccc} {R_{\text{import}}}_{j} & = & \tau ({R_{\text{class}}}_{j}+{R_{\text{c-c}}}_{j})\\ & = & \tau \frac{\theta_{j}\bar{\beta }S_{0}n_{j}+\hat{\delta }\cdot \left({N_{\text{class}}}_{j}-1\right)\cdot \text{Pr}\left(I_{j}⩾I_{\text{limit}}\right)}{\gamma } \end{array}$$ (8)

Based on Eqs (6)–(8), and by considering the effects from initial immunity and loss-of-infectiousness rate, the basic reproduction number of the *j*th age group, ${R_{0}}_{j}$, is derived as follows:

$$\begin{array}{ccc} {R_{0}}_{j} & = & \frac{{R_{\text{class}}}_{j}+{R_{\text{c-c}}}_{j}+{R_{\text{import}}}_{j}}{\left(1-R_{0}\right)\left(1-\eta \right)}\\ & = & \left(1+\tau \right)\cdot \frac{\theta_{j}\bar{\beta }S_{0}n_{j}+\hat{\delta }\cdot \left({N_{\text{class}}}_{j}-1\right)\cdot \text{Pr}\left(I_{j}⩾I_{\text{limit}}\right)}{\gamma \cdot \left(1-R_{0}\right)\left(1-\eta \right)}\;j\in \left\{1,2,...,J\right\} \end{array}$$ (9)

where, ${R_{0}}_{j}$ was the basic reproduction number for the *j*th age group, *θ*_*j*_ was the beta multiplier, and $\bar{\beta }$ was the average transmission rate over a one-year period. *S*_0_ was the initial percentage of Susceptible, and we set *S*_0_ = 34.95%. $\hat{\delta }$ was the fitted class-to-class spread rate. *n*_*i*_ was the number of students per class of the *i*th age group, *N*_class__*j*_ was the number of classes per school for the *j*th age group (Table 1). *I*_*j*_ was the number of secondary infected cases within a class during the infectious period of the initial infected case, *I*_limit_ was the pre-defined outbreak threshold, *I*_limit_ = 5 in the ABM, which was also the trigger of class-to-class transmission. We set *R*_0_, the initial percentage of Recovered, to 65.00%. *η*, the rate of losing transmissibility, to 30%; *τ*, the importing rate, to 5%. *J* was the total number of age groups, and we have *J* = 4 in ABM.

Within the probability term, Pr(*I*_*j*_ ≥ *I*_limit_), we assumed that *I*_*j*_ follows a Binomial distribution, where $I_{j}∼\text{Bino}\left(n=S_{0}n_{j},p=\frac{\theta_{j}\bar{\beta }}{\gamma }\right)$. Poisson distribution was not assumed because *I*_*j*_ should be a finite integer for any given class of ABM.

$R_{0}$ is given by:

$$\begin{array}{c} R_{0}=\sum_{j=1}^{J}p_{j}{R_{0}}_{j} \end{array}$$ (10)

where, ${R_{0}}_{j}$ was the basic reproduction number for the *j*th age group. *p*_*j*_ was the proportion of the model population who belonged to the *j*th age group (Table 1).

### Intervention scenarios

In this study, we compared two scenarios:

- “No intervention” (baseline) scenario
- “School-based vaccination” scenario

“No intervention” scenario is the current status quo in Shenzhen. According to the Shenzhen Education Bureau, specific guidelines for handling varicella outbreaks at schools are not currently available. The “school-based vaccination” scenario is a hypothetical scenario where a school has reported varicella cases beyond the outbreak threshold level, in which case vaccination will be applied to all students within that school, except for the infected or recovered students.

We made simplifying assumptions by considering “single dose” vaccine only. We also ignored “breakthrough cases” where individuals could still get infected after vaccination [3, 10, 11], since they are negligible in numbers. Due to the short time period modelled, we ignored the effects of vaccine waning rate.

## Results

### Model fitting result

**Fig 6:**
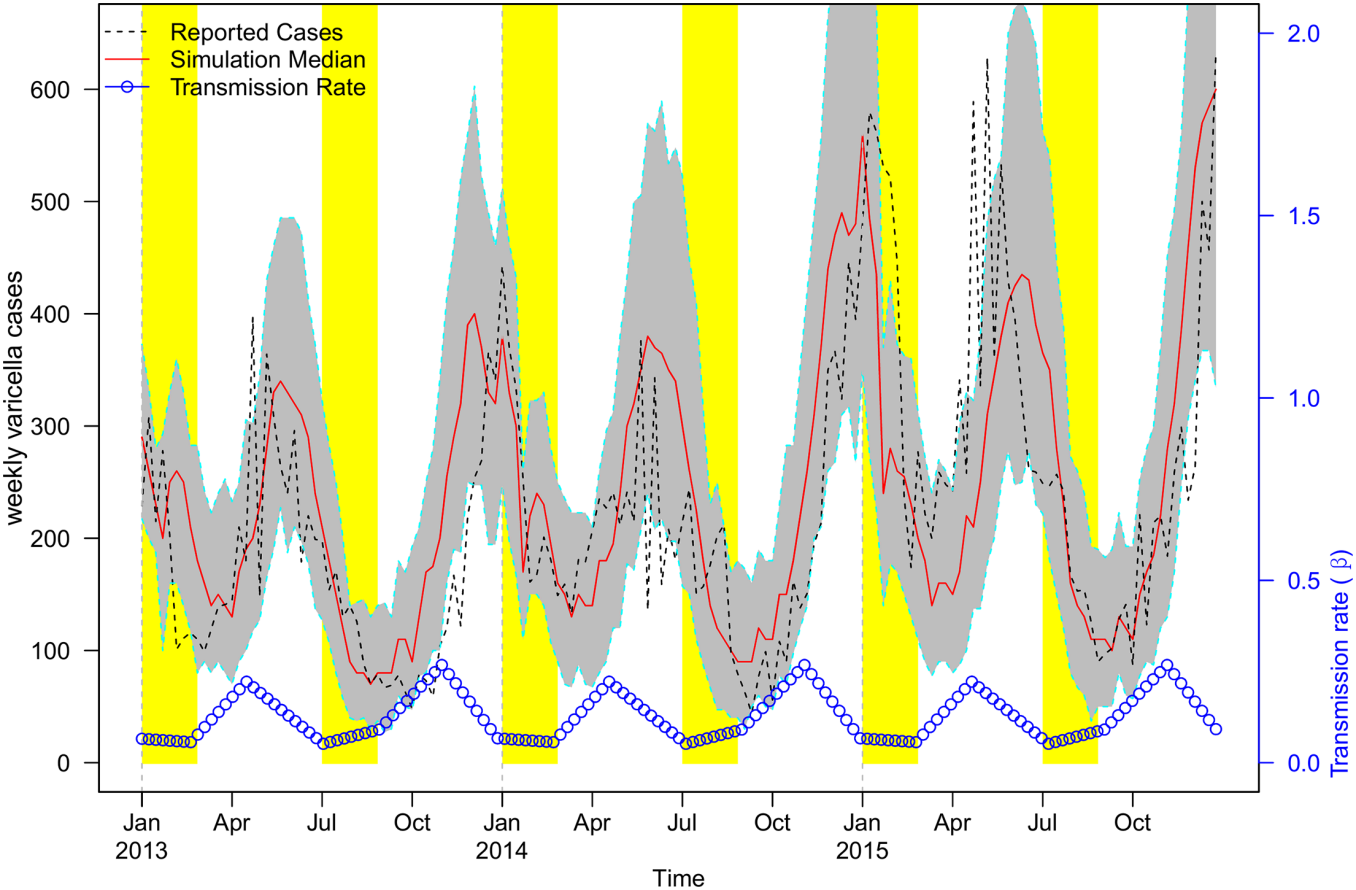
The ABM simulation results of varicella reported cases in Shenzhen from 2013 to 2015. The simulation median is plotted in red, reported cases are in black dashed line, the fitted transmission rate, *β*(*t*), is the blue line at the bottom and the 95% Confidence Interval (C.I.) is in grey. School holidays are shaded in yellow.

We fitted the reported cases on a weekly basis from 2013 to 2015 in Shenzhen, considering a summer wave and a winter wave each year. In Fig 6, the weekly reported cases were compared with the simulation median and their 95% CI from the ABM.

The beta function displays similar patterns in spring and fall semesters, and it appears to be lower during the school holidays than in school terms (see blue-dotted line in Fig 6. More detailed results on the parameter estimation are provided in Table A in S2 File). Thus, our simulated transmission pattern was biologically reasonable [23, 24, 28]. As described above, awareness of disease outbreaks and seasonality could explain the turning point during mid-semester [29].

The best-fitted average class-to-class transmission rate was, *δ* = 1.0 per class⋅week.

### Estimated basic reproduction numbers

The basic reproduction numbers, ${R_{0}}_{j}$, for each age group are shown in Table 4. We could see that there were wide variations in ${R_{0}}_{j}$ among different age groups. This could be due to differences in class size (*N*_*j*,*i*_) and school sizes (as in Table 1), or differences in beta multipliers, *θ*(*a*), among each age group (as in Table 2).

**Table 4:** Table of the basic reproduction numbers, ${R_{0}}_{j}$, for the *j*th age group in Shenzhen.

Age groups (years)	0 - 3	4 - 6	7 - 12	13 - 15
Age group number: *j*	1	2	3	4
${R_{0}}_{j}$	2.4597	6.4670	8.0892	6.6028

Based on Tables 1, 4 and Eq (10), we estimated the overall basic reproduction number in Shenzhen as

$$\begin{array}{c} R_{0}=6.73 \end{array}$$ (11)

Larger $R_{0}$ was found among older age groups, which was consistent with earlier studies [30].

### Impacts of intervention and varying outbreak thresholds on transmission dynamics

**Fig 7:**
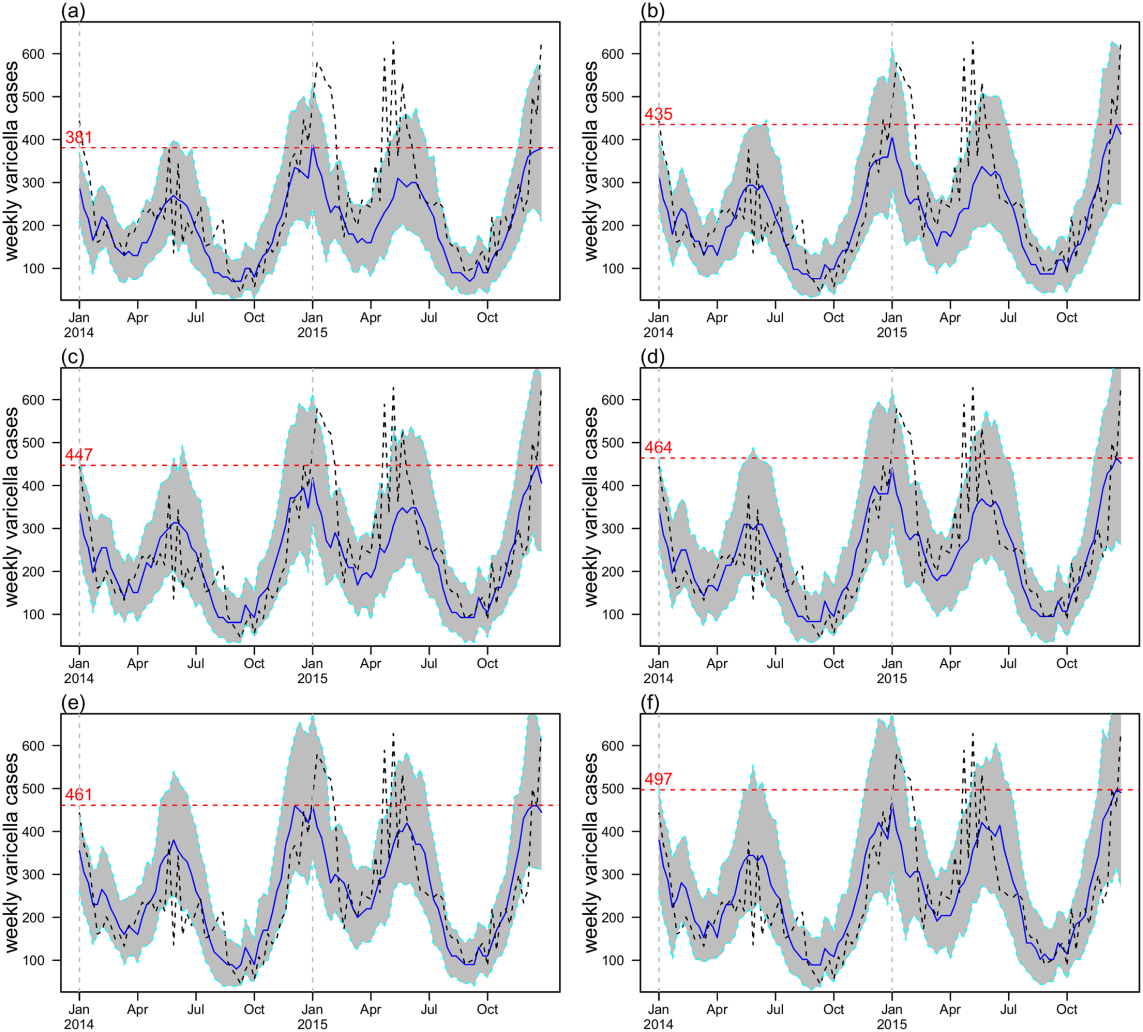
Simulation results with “vaccination” strategy from 2014-2015. The black dashed line is the confirmed cases which could be regarded as the baseline (i.e. no intervention) scenario. Simulation median is plotted in blue with 90% C.I. in grey. Panel (a), (b), (c), (d), (e) and (f) are simulation results with vaccination threshold set to be 5, 6, 7, 8, 9 and 10 (cases per week per school) respectively. The red dashed lines are the maximum weekly varicella cases during the simulation period (blue line), which represents the outbreak size under different outbreak thresholds.

Fig 7 shows the simulation results for the intervention scenario, varying the outbreak threshold that triggered school-based vaccination from five to ten cases. We found that school-based vaccination intervention could effectively prevent large varicella outbreaks. Our results were shown in Table 5. By lowering the outbreak threshold, the school-based vaccination intervention could control the size of outbreaks more tightly. At an outbreak threshold of 5, varicella outbreaks could be reduced by 37% whereas a large school-level outbreak could be effectively controlled with a probability of 97%. (see Table 5 and panel (a) of Fig 7).

**Table 5:** Summary table of the impact of intervention at various outbreak thresholds that triggered school-based vaccination. At each threshold level, we defined the “Maximum outbreak size” as the size of the largest outbreaks from 2014 to 2015, based on the simulation median. “Case reduction” was the percentage of varicella cases reduced due to the school-based vaccination strategy. “Reduction in Size of Outbreaks” was the percentage reduction in the size of the maximum outbreak compared with the baseline scenario. “Proportion of effective control” was the proportion of simulation runs that have simulated cases smaller than the reported cases, a proxy measure that the intervention could effectively bring the number of reported cases under control.

Outbreaks threshold	5	6	7	8	9	10
Maximum outbreaks size	381	435	447	464	461	497
Case reduction	27%	17%	12%	9%	3%	2%
Reduction in size of outbreaks	37%	28%	26%	23%	23%	17%
Proportion of effective control	0.97	0.93	0.89	0.80	0.88	0.75

## Discussion

In this study, we developed a ABM-SEIR model to the reported varicella cases from 2013 to 2015 in Shenzhen. Our model adopted three transmission modes: within-class, class-to-class and out-of-school transmission. We also considered the age structure and an age-specific transmission rate. Our modelling structure is more biologically reasonable than previous studies [10, 12, 13, 18].

The key feature of our model was that the fitting of the transmission rate, *β*(*t*), was strictly referred to as the segment of school terms in Shenzhen. The turning points of the beta function we identified when fitting transmission rate were compared to changes in school terms. Previous studies have applied more flexible time-dependent functions, such as cubic spline functions, to fit the beta function. However, cubic spline functions could possibly result in an over-fitting problem, and in some cases, the trends in transmission rates were not well-observed [31, 32]. We adopted a linear structure in our model fitting, which could offer apparent periodic dynamics in the transmission rate. Our fitted transmission rate function was the same each year, which demonstrates strong seasonality in varicella transmission (Fig 7). The changing dynamics of our fitted beta function were consistent with previous studies [17, 23, 24].

The estimated basic reproduction number, $R_{0}=6.73$, was consistent with previous works [18, 27, 30], suggesting that our model fitting was biologically reasonable. By varying the vaccination thresholds (Table 5 and Fig 7) and re-running the two scenarios, we show that lowering vaccination thresholds could incrementally lead to more effective varicella outbreak control. Our results were both logical and biologically reasonable. We further showed that it was not necessary to conduct school-based vaccination during non-epidemic periods.

Our results add to the varicella modelling literature in two ways. First, our use of ABM-SEIR model considered three levels of transmission that were more realistic than the WAIFW matrix used in previous studies [10–13]. Second, our transmission rate function accounted for major school holidays and provided reasonable model fitness.

Our results of the impact of school-based vaccination (Table 5) were biological reasonable and logical, which provided important theoretical support of disease control decision-making among school population and development of school-based vaccination program. Our model was subject to some limitations. Household transmission, such as those between siblings, as well as reactive behavioral responses during a varicella outbreak, such as contact avoidance, taking medications or seeking clinical treatment, were not considered in our study. These factors could have altered the transmission rate function and the modelling parameters in our ABM-SEIR, and should be a focus in future studies.

## Conclusions

There was a considerable increase in reported varicella cases from 2013 to 2015 in Shenzhen. Our ABM-SEIR was able to fit the two varicella confirmation waves from 2013 to 2015. Our results showed that implementing a school-based vaccination intervention could effectively prevent large outbreaks at various vaccination thresholds. Our study provides important theoretical support for disease control decision making during school outbreaks and the development of a school-based vaccination programme.

## Supporting information

S1 File Steps for parameter estimation. Algorithm A.
(PDF)

S2 File Transmission function estimation results. Table A, Table of *c*_*i*_s estimation ranked by MSE.
(PDF)

S3 File Weekly varicella data. Table A, Table of weekly varicella cases data in Shenzhen from 2010 to 2015.
(PDF)
