# Supplementary material for: Modelling the transmission and control strategies of varicella among school children in Shenzhen, China
Source: PLoS One. 2017 May 18;12(5):e0177514. doi: 10.1371/journal.pone.0177514 (PMC5436677; doi:10.1371/journal.pone.0177514)
Supplement: S1 File — Algorithm A. (PDF) [file pone.0177514.s001.pdf]

Supplementary Materials

Xiujuan Tang[1,☯], Shi Zhao[2,☯], Alice P.Y. Chiu[2,*], Hanwu Ma[1], Xu Xie[1], Shujiang Mei[1], Dongfeng Kong[1], Yanmin Qin[1], Zhigao Chen[1], Xin Wang[1] & Daihai He[2,*]

1 Shenzhen Center for Disease Control and Prevention, Shenzhen, China
2 Department of Applied Mathematics, Hong Kong Polytechnic University, Hong Kong, China

☯These authors contributed equally to this work.
* Corresponding: D.H. `daihai.he@polyu.edu.hk` & A.C. `alice.py.chiu@polyu.edu.hk`

S1 Steps for Parameter Estimation

The steps for parameter estimation are outlined as follows (See Algorithm A). We evenly divide each parameter ($\Theta = [\theta_1, \cdots, \theta_p]^T$) into partitions, which is denoted as total K_i partitions for the ith parameter. At the initial step, we use the current parameter range ($\theta_i \in \left[\theta_i^{(L)}, \theta_i^{(U)}\right]$. For each of the total $\prod_{i=1}^{p} K_i$ combinations of parameters (θ_is), the simulation fitting is being run for N times, where N is a sufficiently large number. We compute the mean squared error for each run, i.e. $\widetilde{\text{mse}}_j$ for the jth combination of parameters. After simulating all $\prod_{i=1}^{p} K_i$ combinations of parameters, we identify the jth parameters combination $\widehat{\Theta} = [\theta_{1_{q_1}}, \cdots, \theta_{p_{q_p}}]^T$ that has the smallest $\widetilde{\text{mse}}_j$. (denoted as ($\text{MSE}_{\min} = \min\left\{\widetilde{\text{mse}}_j \middle| j \in \{1, \ldots, \prod_{i=1}^{p} K_i\}\right\}$)). Parameter ranges are then updated according to the current best-fitted parameter combination ($\widehat{\Theta}$) and the updated ranges are re-applied in the next step. When an updated range are within the acceptable error level (denoted as ε_i for the ith parameter), this parameter estimate is considered as a “acceptable”. It is then output as a parameter estimate and is used to infer the rest of parameters.

**Algorithm A**	
**input:**	parameter, $\Theta = [\theta_1, \cdots, \theta_p]^T$; runs for simulation, N; set of parameter index, $\mathbf{P} = \{1, \ldots, p\}$;<br>ranges of parameters, $\theta_i \in \left[\theta_i^{(L)}, \theta_i^{(U)}\right]$; number of partitions for parameters, $K_i \geqslant 2$;<br>error level, $0 < \varepsilon_i < (\theta_i^{(U)} - \theta_i^{(L)})$; estimator, $\widehat{\Theta} = [\widehat{\theta}_1, \cdots, \widehat{\theta}_p]^T$
**Do {**	
**For** $i \in \mathbf{P}$	
	$\{\Theta_i\} = \left\{\theta_{i1} = \theta_i^{(L)}, \theta_{i2}, \ldots, \theta_{iK_i} = \theta_i^{(U)}\right\}$, with K_i partitions evenly distributed in $\left[\theta_i^{(L)}, \theta_i^{(U)}\right]$<br>record $\{\Theta_i\}$
**end For**	
**For** each Θ	*(where, $\theta_i \in \{\Theta_i\}$ with $i \in \boldsymbol{P}$ and there are $\prod_{i=1}^{p} K_i$ combinations of $\theta_i s$)*<br>given Θ_j, where $j \in \{1, \ldots, \prod_{i=1}^{p} K_i\}$, do N runs of simulation<br>find the median of mean squared error, $\widetilde{\text{mse}}_j$, from N runs of simulation<br>record $(\Theta_j, \widetilde{\text{mse}}_j)$, with $j \in \{1, \ldots, \prod_{i=1}^{p} K_i\}$
**end For**	
**Find** MSE_{min}	
	MSE_{min} = minimum of all $\widetilde{\text{mse}}_j$s, with $j \in \{1, \ldots, \prod_{i=1}^{p} K_i\}$<br>reset $\widehat{\Theta} = [\theta_{1q_1}, \cdots, \theta_{p_{q_p}}]^T$ for the combination of parameter that have achieved MSE_{min}
**end Find**	
**Set** parameter range	
**For** $i \in \mathbf{P}$	
	update $\theta_i^{(L)} = \theta_{i(q_i-1)}$ and $\theta_i^{(U)} = \theta_{i(q_i+1)}$, where $q_i \in \{2, \ldots, (K_i - 1)\}$
**If** $\varepsilon_i \geqslant (\theta_i^{(U)} - \theta_i^{(L)})$	
	$\mathbf{P} = \mathbf{P}/\{i\}$
**end If**	
**end For**	
	record $\theta_i^{(L)}$ and $\theta_i^{(U)}$, with $i \in \mathbf{P}$
**end Set**	
**} While** $\left(\mathbf{P} \neq \varnothing\right)$	
**output:**	estimator: $\widehat{\Theta}$
