# Supplementary material for: Modelling the transmission and control strategies of varicella among school children in Shenzhen, China
Source: PLoS One. 2017 May 18;12(5):e0177514. doi: 10.1371/journal.pone.0177514 (PMC5436677; doi:10.1371/journal.pone.0177514)
Supplement: S2 File — Table A, Table of cis estimation ranked by MSE. (PDF) [file pone.0177514.s002.pdf]

Supplementary Materials

Xiujuan Tang[1,◕], Shi Zhao[2,◕], Alice P.Y. Chiu[2,*], Hanwu Ma[1], Xu Xie[1], Shujiang Mei[1], Dongfeng Kong[1], Yanmin Qin[1], Zhigao Chen[1], Xin Wang[1] & Daihai He[2,*]

1 Shenzhen Center for Disease Control and Prevention, Shenzhen, China
2 Department of Applied Mathematics, Hong Kong Polytechnic University, Hong Kong, China

◕These authors contributed equally to this work.
* Corresponding: D.H. daihai.he@polyu.edu.hk & A.C. alice.py.chiu@polyu.edu.hk

S2 Transmission Function Estimation Results

In Table A, we list the six c_is' combinations with the smallest MSE. The best-fitted model is displayed at the top row which has a MSE of 12,875.

Table A. Table of c_is estimation ranked by MSE. c_i represents the constant term of transmission rate $\beta(t_{\text{week}})$

Week segment (i)	1	2	3	4	5	6	
Week number (t_i)	1	9	17	27	36	44	MSE
1	0.067	0.057	0.222	0.051	0.091	0.268	12875
2	0.068	0.056	0.222	0.052	0.092	0.255	12991
3	0.068	0.057	0.226	0.052	0.091	0.259	13011
4	0.068	0.056	0.223	0.052	0.089	0.259	13694
5	0.065	0.057	0.224	0.050	0.090	0.283	13732
6	0.069	0.058	0.224	0.049	0.090	0.279	13962
