# Supplementary material for: Modelling the transmission and control strategies of varicella among school children in Shenzhen, China
Source: PLoS One. 2017 May 18;12(5):e0177514. doi: 10.1371/journal.pone.0177514 (PMC5436677; doi:10.1371/journal.pone.0177514)
Supplement: S3 File — Table A, Table of weekly varicella cases data in Shenzhen from 2010 to 2015. (PDF) [file pone.0177514.s003.pdf]

Supplementary Materials

Xiujuan Tang[1,☯], Shi Zhao[2,☯], Alice P.Y. Chiu[2,*], Hanwu Ma[1], Xu Xie[1], Shujiang Mei[1], Dongfeng Kong[1], Yanmin Qin[1], Zhigao Chen[1], Xin Wang[1] & Daihai He[2,*]

1 Shenzhen Center for Disease Control and Prevention, Shenzhen, China
2 Department of Applied Mathematics, Hong Kong Polytechnic University, Hong Kong, China

☯These authors contributed equally to this work.
* Corresponding: D.H. `daihai.he@polyu.edu.hk` & A.C. `alice.py.chiu@polyu.edu.hk`

S3 Weekly Varicella Data

In Table A, we list the weekly varicella cases data in Shenzhen from 2010 to 2015.

Table A. Table of weekly varicella cases data in Shenzhen from 2010 to 2015

Week	2013	2014	2015
1	229	443	482
2	308	370	580
3	215	333	562
4	278	253	531
5	203	161	522
6	101	168	445
7	111	201	237
8	116	181	174
9	110	149	277
10	99	159	222
11	119	133	200
12	139	181	259
13	141	179	248
14	144	207	242
15	210	235	341
16	180	227	258
17	397	241	589
18	148	212	336
19	364	241	628
20	309	215	360
21	264	376	533
22	240	136	429
23	296	343	395
24	179	159	324
25	220	208	261
26	199	181	259
27	198	215	249
28	154	244	247
29	171	151	257
30	130	157	242
31	140	176	167
32	126	202	153
33	88	212	154
34	70	98	125
35	79	79	90
36	67	63	97
37	69	44	102
38	78	77	128
39	63	99	141
40	48	56	87
41	79	108	221
42	73	90	123
43	59	162	214
44	104	138	220
45	120	151	184
46	167	195	259
47	122	213	299
48	219	350	234
49	254	367	264
50	269	324	500
51	366	446	457
52	341	395	634
